# A Novel Molecular Complex Expressed on Immature B Cells: A Possible Role in T Cell-Independent B Cell Development

**DOI:** 10.1155/1996/21973

**Published:** 1996

**Authors:** Philip J. Griebel, Paolo Ghia, Ulf Grawunder, Giorgio Ferrari

**Affiliations:** 1 Veterinary Infectious Disease Organization University of Saskatchewan 120-Veterinary Road Saskatoon SK S7N 5E3 Canada; 2 Basel Institute for Immunology 487-Grenzacherstrasse Postfach Basel CH-4005 Switzerland; 3 Washington University School of Medicine Division of Molecular Oncology St. Louis Missouri USA

**Keywords:** B cell, monoclonal antibody, Peyer's patch, pro-B cell, sheep

## Abstract

To identify surface molecules that may play a role in regulating ileal Peyer's patch (PP) B cell growth, we generated monoclonal antibodies (mAbs) and then selected them for a unique reactivity with ileal PP B cells. Flow cytometric analysis identified a mAb (SIC4.8R) that labeled 97% of ileal and 50–60% of jejunal PP sIgM^+^B cells. SIC4.8R also labeled a subpopulation of cortical thymocytes but few B or T cells in other lymphoid tissues, including bone marrow. Immunohistochemistry revealed intense SIC4.8R staining of B cells in the cortex of ileal PP follicles. SIC4.8R also labeled bovine PP B cells, a murine pro-B cell line, and pre-B cells in human bone marrow. Protein chemistry revealed that a structurally similar molecular complex was expressed on sheep ileal PP B cells and thymocytes and murine pro-B cells. Addition of soluble SIC4.8R to cultured ileal PP B cells reduced apoptotic cell death, elevated proliferative responses, partially inhibited anti-Ig-induced cell death, and induced IL-4 responsiveness. In contrast, soluble SIC4.8R had an antiproliferative effect on a mouse pro-B cell line. Finally, SIC4.8R labeling declined following the stimulation of ileal PP B cells with CD40 ligand. In conclusion, the present investigation determined that SIC4.8R identified a novel molecular complex that is expressed at several stages of T cell-independent B cell development in a variety of mammalian species. This observation confirmed that PP B cells are developmentally distinct from other B cell populations in sheep and suggested that the bone marrow may not be a site of B lymphopoiesis in young lambs.


*Development Immunology*, 1996, Vol 5, pp. 67–78
Reprints available directly from the publisher
Photocopying permitted by license only

© 1996 OPA (Overseas Publishers Association)
Amsterdam B.V. Published in The Netherlands
by Harwood Academic Publishers GmbH
Printed in Singapore

### A Novel Molecular Complex Expressed On Immature B Cells: A Possible Role in T Cell-Independent B Cell Development

PHILIP J. GRIEBEL,* PAOLO GHIA,[†] ULF GRAWUNDER,[‡] and GIORGIO FERRARI[†]

[†]*Basel Institute for Immunology, 487-Grenzacherstrasse, Postfach, CH-4005, Basel, Switzerland*
[‡]*Washington University School of Medicine, Division of Molecular Oncology, St. Louis, Missouri, USA*

To identify surface molecules that may play a role in regulating ileal Peyer's patch (PP) B cell growth, we generated monoclonal antibodies (mAbs) and then selected them for a unique reactivity with ileal PP B cells. Flow cytometric analysis identified a mAb (SIC4.8R) that labeled 97% of ileal and 50–60% of jejunal PP sIgM⁺B cells. SIC4.8R also labeled a subpopulation of cortical thymocytes but few B or T cells in other lymphoid tissues, including bone marrow. Immunohistochemistry revealed intense SIC4.8R staining of B cells in the cortex of ileal PP follicles. SIC4.8R also labeled bovine PP B cells, a murine pro-B cell line, and pre-B cells in human bone marrow. Protein chemistry revealed that a structurally similar molecular complex was expressed on sheep ileal PP B cells and thymocytes and murine pro-B cells. Addition of soluble SIC4.8R to cultured ileal PP B cells reduced apoptotic cell death, elevated proliferative responses, partially inhibited anti-Ig-induced cell death, and induced IL-4 responsiveness. In contrast, soluble SIC4.8R had an antiproliferative effect on a mouse pro-B cell line. Finally, SIC4.8R labeling declined following the stimulation of ileal PP B cells with CD40 ligand. In conclusion, the present investigation determined that SIC4.8R identified a novel molecular complex that is expressed at several stages of T cell-independent B cell development in a variety of mammalian species. This observation confirmed that PP B cells are developmentally distinct from other B cell populations in sheep and suggested that the bone marrow may not be a site of B lymphopoiesis in young lambs.

KEYWORDS: B cell, monoclonal antibody, Peyer's patch, pro-B cell, sheep.

### INTRODUCTION

For many mammalian species, the gut-associated lymphoid tissues, including Peyer's patches (PP), provide an unique and essential environment for B cell development (Cooper and Lawton, 1972; Chapman et al., 1974; Reynolds and Morris, 1983). This is evident for the ileal PP of sheep, which functions as a primary lymphoid tissue for B cell development with antigen-independent and T cell-independent diversification of the Ig receptor repertoire (Reynaud et al., 1995). Furthermore, numerous *in vivo* and *in vitro* investigations have revealed that B cells present within lymphoid follicles of the ileal PP (iPfB cells) of sheep are developmentally and phenotypically distinct from B cells in other lymphoid tissues (Reynolds, 1986; Hein et al., 1989; Griebel et al., 1991, 1992a).

B cell production in the ileal PP begins during fetal development and continues at approximately the same rate until lambs reach sexual maturity (Reynolds, 1987). During this time, the mitotic index within ileal PP lymphoid follicles is four to five times greater than that of the thymus. However, approximately 95% of the iPfB cells produced then die by apoptosis, which suggests that B cell development is closely regulated by a number of soluble factors or cell-cell interactions. (Reynolds, 1986;

*Corresponding author: Present address: Veterinary Infectious Disease Organization, University of Saskatchewan, 120-Veterinary Road, Saskatoon, SK, Canada S7N 5E3.

Motyka and Reynolds, 1991). The proliferation of iPfB cells can be inhibited by either anti-Ig treatment *in vitro* (Griebel et al., 1991) or corticosteroid treatment *in vivo* (Griebel et al., 1996). Furthermore, iPfB-cell proliferation can be sustained in culture by a direct B cell-stromal cell interaction (Griebel and Ferrari, 1994) but not an interaction with CD40 ligand (Griebel and Ferrari, 1995). Thus, B cell production in the ileal PP appears to be T cell-independent and may be sustained by a variety of B cell-stromal cell interactions (Griebel et al., 1993).

Previous investigations revealed that many surface molecules present on mature B cells of sheep were absent from the immature iPfB cells (Hein et al., 1989; Griebel et al., 1992b). Therefore, we hypothesized that the unique physiology and function of B cells in the ileal PP would be reflected in the expression of a distinctive array of surface molecules. Furthermore, we postulated that some of these molecules may be involved in regulating iPfB cell proliferation and differentiation. To test this hypothesis, we generated a panel of monoclonal antibodies (mAbs) that were screened for a specific reactivity with iPfB cells. One such mAb was identified and then tested *in vitro* to determine if it identified a molecule that could transduce a signal(s) that influenced iPfB cell growth.

### RESULTS

### Selection of iPfB Cell-Specific Monoclonal Antibodies

A total of 186 hybridomas were screened with flow cytometry and 16 mAbs were identified that reacted with iPfB cells but not PBM cells. IHC revealed that 12 of the mAbs selected reacted with antigens that were also present in mesenchymal cells or mucosal epithelial cells. One mAb (SIC4.8R; IgM isotype) reacted with a surface molecule that was expressed at a high level on most sIgM$^+$ iPfB cells (Fig. 1a; Table 1), but a minor subpopulation (<10%) of B cells in blood (Fig. 1c; Table 1). SIC4.8R also labeled a subpopulation of sIgM$^+$ B cells in jejunal PP follicles (Fig. 1b; Table 1) and minor B cell subpopulations in the MLN and efferent lymph from the prescapular lymph node (Table 1). SIC4.8R reactivity was not restricted to the B cell lineage, but also included a subpopulation of blood T cells (Fig. 1g) and cortical CD5$^{lo}$) thymocytes (Fig. 1h). IHC staining of lamb tissues with SIC4.8R revealed that lymphocyte clusters in the cortex of ileal PP follicles expressed a high level of antigen (Figs 2A and B). In contrast, intense staining by SIC4.8R was observed for most lymphocytes within the follicles of fetal jejunal and ileal

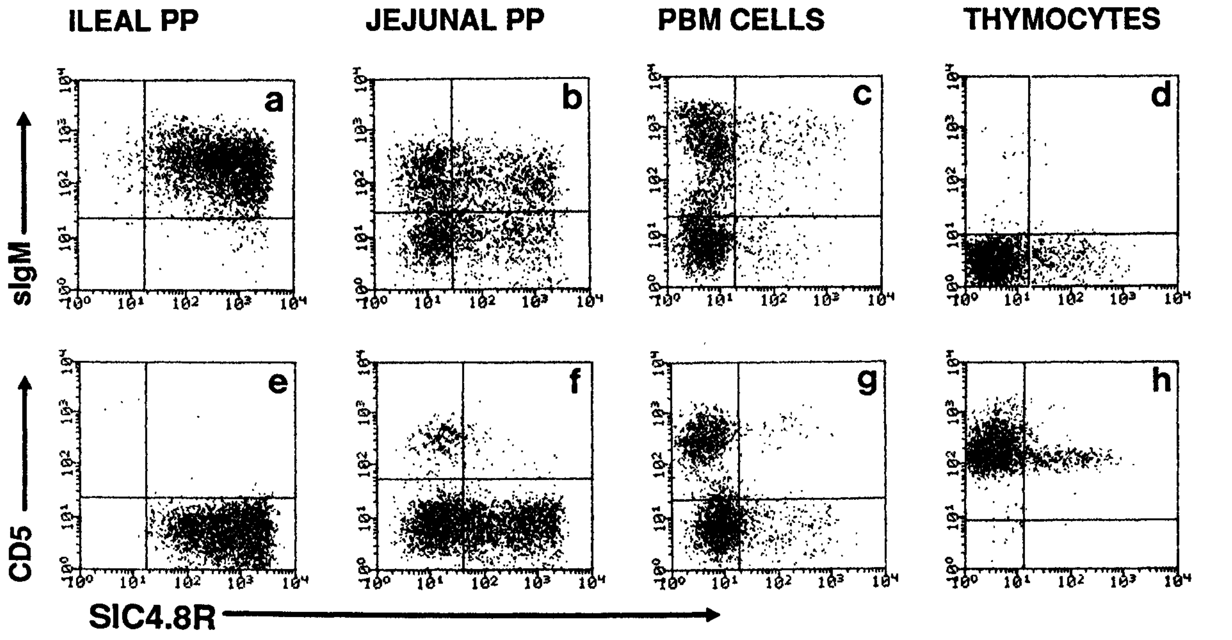

FIGURE 1. SIC4.8R labels most sIgM$^+$ B cells in ileal PP follicles, a subpopulation of sIgM$^+$ B cells in jejunal PP follicles but few sIgM$^+$ B cells from the blood (PBM cells) of young lambs. SIC4.8R also labeled a minor subpopulation of T cells (CD5$^+$) in all lymphoid tissues examined, including the thymus (CD5$^{lo}$).

PP (data not shown). This was consistent with the flow cytometric data, which revealed that most sIgM+ B cells in the ileal and jejunal PP of a 144-day fetus (Figs. 3a and b) were labeled at a high level. Few T cells in the fetal PP were labeled (Figs 3e and f), but some T cells in blood and thymus were labeled (Figs 3g and h). These analyses indicated that SIC4.8R identified a molecule expressed on immature lymphocytes of both the B (iPfB cells) and T cell (cortical thymocytes; $CD5^{lo}$) lineage, but not lymphoid, myeloid, or erythroid lineages in the bone marrow (Figs 4a and b).

### Cross-Species Reactivity of SIC4.8R Monoclonal Antibody

To further characterize the specificity of the SIC4.8R mAb, its reactivity with leukocytes from a variety of species was analyzed. A strong reaction was observed with B cells isolated from the ileal and jejunal PP, but not PBM cells of young calves (data not shown). This indicated that the pattern of SIC4.8R labeling was conserved among ruminants. A more remarkable conservation was suggested by SIC4.8R labeling of a murine pro-B cell line (Fig. 4d). This RAG-2 gene-deleted pro-B-cell line was chosen because it did not express endogenous IgM (Fig. 4e). Furthermore, co-staining for SIC4.8R and a number of pro-B-cell surface molecules, including heat-stable antigen (HSA; Figs 4e and f), CD25, CD43, and IL-7 receptor, revealed that the SIC4.8R molecule was

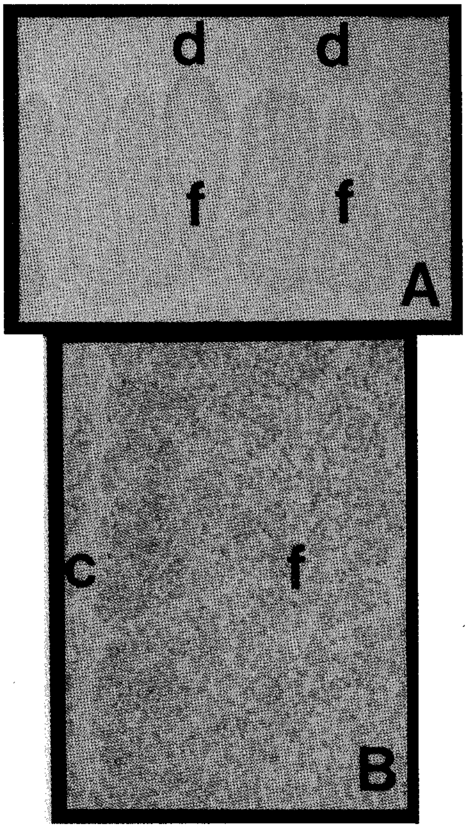

FIGURE 2. SIC4.8R specifically stained lymphocytes in lymphoid follicles of the ileal PP of a lamb. (A) Immunoperoxidase staining was most intense in the cortex of the follicles (f) with no visible staining in the dome (d) region. Magnification: ×25. (B) High magnification (×250) of the ileal PP follicle (f) clearly shows that clusters of cells adjacent to the follice capsule (c) stain intensely with SIC4.8R.

TABLE 1
Labeling of T and B lymphocytes Populations by SIC4.8R

| | Percent positive cells[a] | | | | |
|---|---|---|---|---|---|
| Cell population | Total SIC4.8R+ | sIGM+ SIC4.8R+ | sIgM+ SIC4.8R− | CD5+ SIC4.8R+ | CD5+ SIC4.8R− |
| Ileal PP[b] | 96.6 ± 1.3 | 94.6 ± 1.8 | 2.8 ± 0.6 | 0.04 ± 0.02 | 0.22 ± 0.05 |
| Jejunal PP[b] | 47.2 ± 3.4 | 27.2 ± 2.9 | 26.8 ± 2.4 | 1.5 ± 0.3 | 9.9 ± 1.1 |
| MLN[c] | 14.5 ± 2.3 | 8.8 ± 1.2 | 21.0 ± 2.9 | 7.5 ± 2.2 | 59.1 ± 3.4 |
| PBM Cells[d] | 9.2 ± 2.7 | 4.8 ± 0.6 | 44.3 ± 4.1 | 3.5 ± 0.4 | 38.4 ± 2.8 |
| Eff. Lymph[e] | 8.8 ± 0.9 | 1.4 ± 0.6 | 13.3 ± 1.8 | 6.8 ± 1.8 | 78.4 ± 3.8 |
| Bone marrow[f] | 4.3 ± 1.1 | 2.1 ± 0.7 | 32.5 ± 4.4 | 1.8 ± 0.6 | 34.1 ± 3.7 |
| Thymocytes | 22.4 ± 6.2 | 0.05 ± 0.03 | 0.6 ± 0.2 | 21.4 ± 7.4 | 77.0 ± 6.9 |

[a]Data presented are the mean ± S.D. of values for cells isolated from 4, 6–10-week-old lambs.
[b]Cell suspensions prepared from PP lymphoid follicles.
[c]MLN: mesenteric lymph node.
[d]PBM: peripheral blood mononuclear cells.
[e]Lymph draining the prescapular lymph nodes of 1–2-year-old sheep.
[f]Total SIC4.8R+ cells included an analysis of erythroid, myeloid, and lymphoid populations in bone marrow cells.

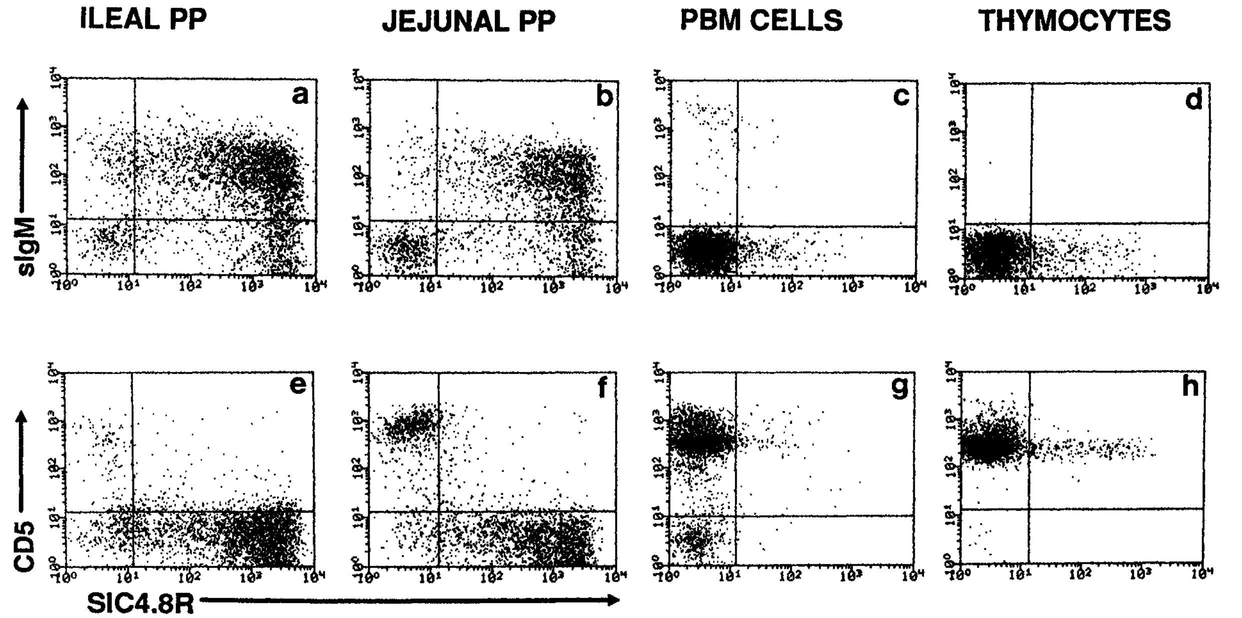

FIGURE 3. SIC4.8R labels most sIgM$^+$B cells in fetal ileal and jejunal PP follicles but subpopulations of sIgM SIC4.8R$^+$B cells were also evident. Few sIgM$^+$B cells from the blood (PBM cells) were labeled but a subpopulation of T cells (CD5$^+$) in fetal blood and thymus (CD5$^{lo}$) were SIC4.8R$^+$.

distinct from these known pro-B-cell surface molecules. Further conservation of the SIC4.8R molecule was suggested by the specific labeling of human CD10$^+$CD19$^+$ pre-B cells but not mature B cells in bone marrow or blood (Table 2). However, SIC4.8R reactivity also included a minor subpopulation of NK cells (Table 2). Finally, conservation of the SIC4.8R reactivity did not extend to chickens because neither bursal B cells or blood leukocytes were labeled by this mAb (data not shown).

### Protein Chemistry of the SIC4.8R Molecular Complex

The extensive cross-species activity of SIC4.8R mAb suggested that either a single epitope was highly conserved or that a fortuitous cross-reactivity occurred among distinct molecules. Protein chemistry was used to determine if a structurally similar protein was identified by SIC4.8R on both sheep and mouse cells. The SIC4.8R mAb precipitated a molecular complex from sheep iPfB cells that consisted of multiple proteins that were visible under reducing conditions (Fig. 5). Following iPfB cell lysis with Brij, the SIC4.8R mAb specifically precipitated several proteins but the three bands indicated at 53, 55, and 75 kD (Fig. 5, lanes 1 and 2; reduced) were the only proteins observed following lysis with NP-40 and digitonin (data not shown). Three proteins of similar molecular weight were seen with lysates of sheep thymocytes but the 75-kD band often gave a much stronger signal than the 53- and 55-kD proteins (data not shown). SIC4.8R also precipitated a multimeric protein complex from surface-

TABLE 2
SIC4.8R Labeling of Lymphocyte Subpopulations from Human Bone Marrow and Blood

| Cell population | Percent positive cells[a] Bone marrow[b] | PBM cells[c] |
|---|---|---|
| Progenitor B cells (CD10$^+$, CD19$^+$, CD34$^+$, TdT$^+$) | <5 | N.D.[d] |
| Precursor B cells (CD10$^+$, CD19$^+$, Cyto-μ$^+$) | 60–75 | N.D. |
| Immature B cells (CD10$^+$, CD19$^+$, sIgM$^+$, sIgD$^-$) | <5 | N.D. |
| Mature B cells (CD10$^-$, CD19$^+$, sIgM$^+$, sIgD$^+$) | <0.1 | <0.1 |
| γδ T cells | <0.1 | <0.1 |
| αβ T cells | <0.1 | <0.1 |
| NK-cells (CD2$^+$, CD56$^+$) | 5–10 | 6–10 |

[a]SIC4.8R$^+$ cells expressed as a percentage of each subpopulation. Data presented as the range of values.

[b]Bone marrow biopsies from 4, 3–11-year-old children.

[c]Blood collected from 3 clinically normal individuals over 18 years of age.

[d]N.D.: not determined.

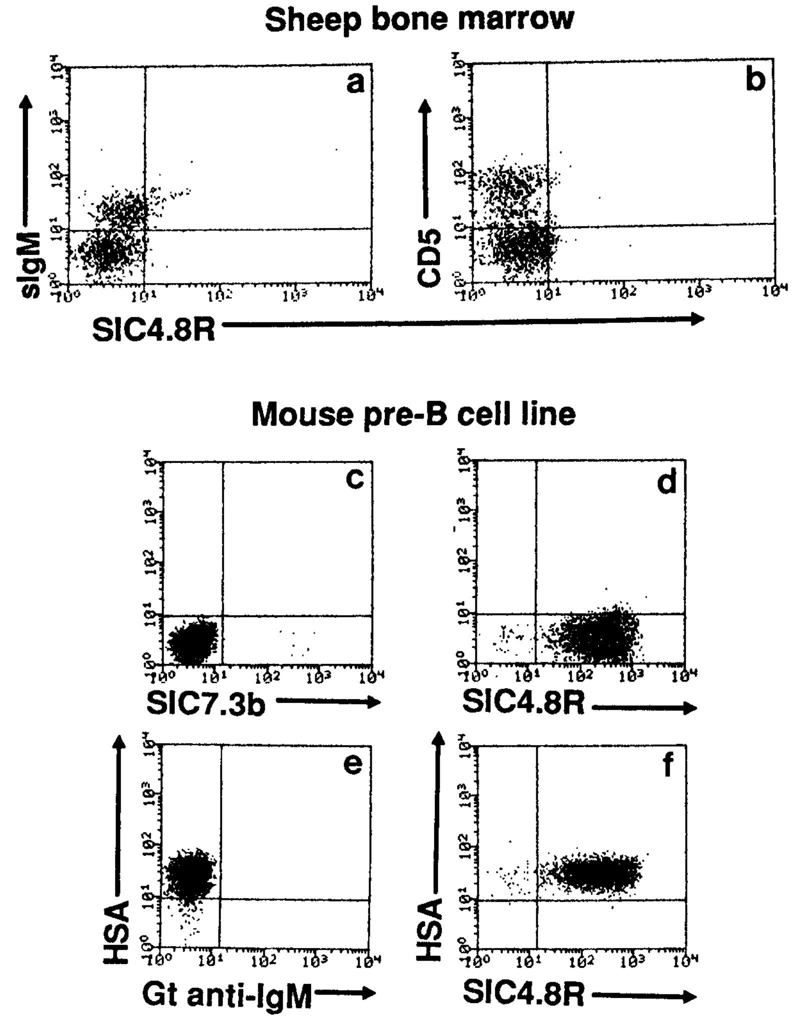

FIGURE 4. SIC4.8R labeling of lymphoid cells in the bone marrow of a lamb revealed few positive cells among the sIgM+ B cells (a), CD5+ T cells (b), or other lymphoid-lineage cells. The lymphoid (R1; 6.3% nucleated cells), erythroid (R2; 27.9% nucleated cells), and myeloid (R3; 24.0% nucleated cells) bone marrow cells were analyzed but no SIC4.8R+ cells were detected in either R2 or R3. In contrast, a pro-B cell line, established from a RAG-2 gene-deleted/*bcl*-2 transgenic mouse, labeled strongly with SIC4.8R (d) but not SIC7.3b, an isotype-matched mAb (c). Co-labeling of mouse pro-B cells with anti-HSA and SIC4.8R indicated that these were distinct molecules (f).

biotinylated, Brij-lysed mouse pro-B cells (Fig. 5; lane 6). This complex consisted of at least three proteins with clearly visible bands at 53, 55, and 75 kD. A number of other bands appeared to be specific but were faintly visible and were not seen when NP-40 or digitonin were used for cell lysis (data not shown). In conclusion, protein chemistry clearly showed that the SIC4.8R mAb reacted with a multimeric complex on both sheep iPfB cells and mouse pro-B cells. The number of proteins in this complex varied with the detergent used for cell lysis, which suggests that this complex included non covalently linked proteins. This was most evident with sheep iPfB cells where a greater number of proteins were precipitated following Brij lysis and some of these bands were similar in size to proteins that were precipitated by an anti-IgM reagent (Fig. 5: lanes 1 and 3; reduced).

### B Cell Growth Is Altered by Soluble SIC4.8R Monoclonal Antibody

To determine if the SIC4.8R molecular complex could function to regulate B cell growth, we assayed the effects of soluble mAb on cultured iPfB cells. IPfB cells rapidly undergo apoptotic cell death during culture (Motyka and Reynolds, 1991) and this

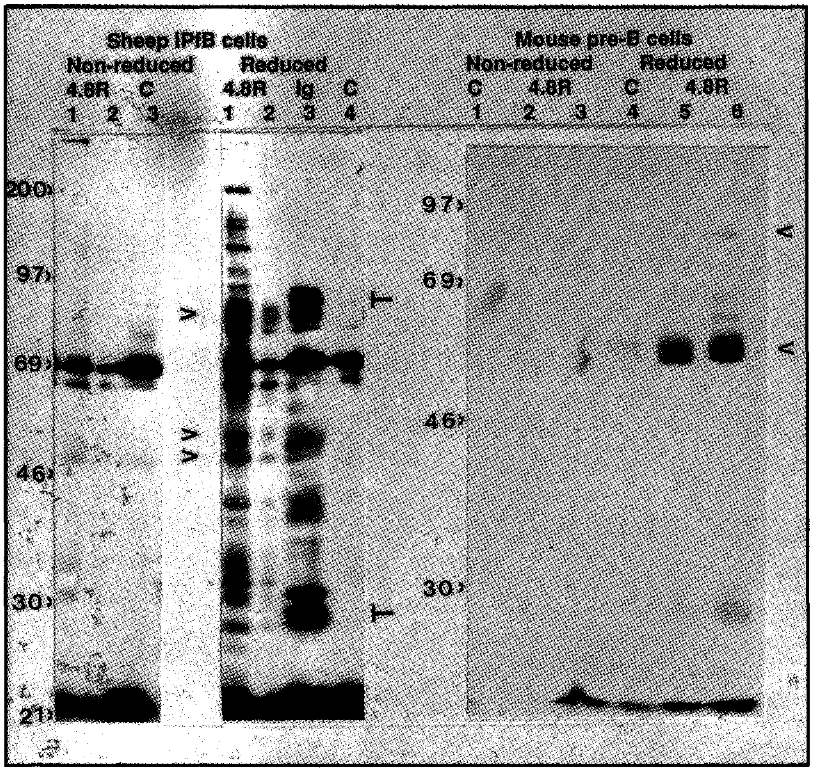

FIGURE 5. Immunoprecipitation of biotinylated surface proteins from sheep iPfB cells and a mouse pro-B cell line, following lysis with Brij, revealed that SIC4.8R reacted with a multimeric protein complex. iPfB cells, non-reduced: Lane 1: Lysate from 5 x $10^6$ iPfB cells reacted with SIC4.8R; lane 2: Lysate from 2 x $10^6$ iPfB cells reacted with SIC4.8R; lane 3: Lysate from 5 x $10^6$ iPfB cells reacted with SIC7.3b (IgM isotype control). No specific proteins were visible under nonreducing conditions. iPfB cells, reduced: Lane 1: Lysate from 5 x $10^6$ iPfB cells reacted with SIC4.8R. Numerous specific bands were visible and bands at 52, 55, and 75 kD are indicated (>) because these bands were also visible in lane 2 where lysate from 2 x $10^6$ iPfB cells was reacted with SIC4.8R. Lane 3: Lysate from 5 x $10^6$ cells reacted with rabbit anti-sheep IgM revealed a heterogeneous Ig heavy chain (80 kD; ⊥), Ig light chain (28 kD; ⊥), and at least 2 other Ig-associated proteins between 30–50 kD. Lane 4: Lysate from 5 x $10^6$ iPfB cells reacted with the SIC7.3b mAb (IgM isotype control). Mouse pro-B cells, non-reduced: Lane 1: Lysate from 5 x $10^6$ pro-B cells reacted with SIC7.3b mAb (IgM isotype control). Lane 2: Lysate from 2 x $10^6$ pro-B cells reacted with SIC4.8R, Lane 3: Lysate from 5 x $10^6$ pro-B cells reacted with SIC4.8R, No specific bands were visible under nonreducing conditions. Mouse pro-B cells, reduced: Lane 4: Lysate from 5 x $10^6$ pro-B cells reacted with SIC7.3b mAb (IgM isotype control). Lane 5: Lysate from 2 x $10^6$ pro-B cells reacted with SIC4.8R. Specific protein bands were visible at 52 and 55 kD (<). Lane 6: Lysate from 5 x $10^6$ reacted with SIC4.8R. A third protein band was then visible at 75 kD (<) with faint shadows between 60–60 kD and below 30 kD.

apoptotic cell death can be quantitated by a flow cytometric analysis of PI incorporation and FALS (Griebel and Ferrari, 1995). Following a 24-hr incubation with 10 μg/ml of the purified, irrelevant SIC7.3b mAb (IgM isotype), less than 35% of iPfB cells remained viable and many cells were $PI^+FALS^{lo}$ (Fig. 6a). A 24-hr co-culture with mCD40L/J558L cells was included as a positive control to demonstrate a clear reduction in iPfB cell apoptotic cell death (Fig. 6b; Griebel and Ferrari, 1995). Following a 24-hr incubation with 10 μg/ml of purified SIC4.8R mAb, there was a two-fold increase in viable iPfB cell number that correlated with a marked reduction in the percentage of $PI^+FALS^{lo}$ cells (Fig. 6c). Thus, soluble SIC4.8R acted as a ligand that induced a signal(s) that blocked apoptotic cell death and supported an increased level of iPfB cell proliferation (Fig. 7a).

Previous investigations determined that ligating surface (s)IgM on iPfB cells with soluble antibody (anti-Ig) resulted in cell death (Griebel et al., 1991). However, anti-Ig activity was inhibited by either CD40 co-signaling (Griebel and Ferrari, 1995) or an interaction between stromal cells and iPfB cells (Griebel and Ferrari, 1994). Therefore, we investigated the possibility that co-signaling by the SIC4.8 complex would also alter the effect of anti-Ig on iPfB cells. The addition of soluble SIC4.8R mAb reduced the anti-mitotic effect of anti-Ig, and with the addition of both IL-4 and soluble SIC4.8R mAb, the inhibitory activity of anti-Ig was reduced by approximately 50% (Fig. 7a). Therefore, mAb binding to the

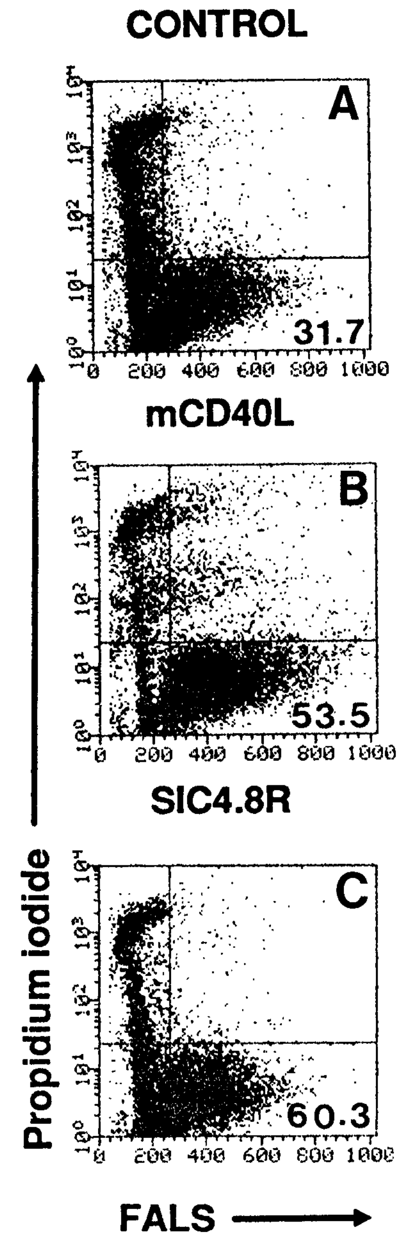

FIGURE 6. Soluble SIC4.8R inhibits iPfB cell apoptotic cell death (A) IPfB cells cultured for 24 hr in medium (control; 10 µg/ml SIC7.3b mAb) undergo extensive cell death by apoptosis (PI$^+$ FALS$^{lo}$) with few viable cells surviving in culture (31.7% PI$^-$ FALS$^{hi}$). (B) Co-culture with mCD40L/J558L cells for 24-hr reduced iPfB-cell apoptotic cell death and increased cell viability (53.5% PI$^-$ FALS$^{hi}$). (C) The addition of 10 µg/ml soluble SIC4.8R for 24-hr reduced iPfB-cell apoptotic cell death and doubled cell viability (60.3% PI$^-$ FALS$^{hi}$) relative to the control culture.

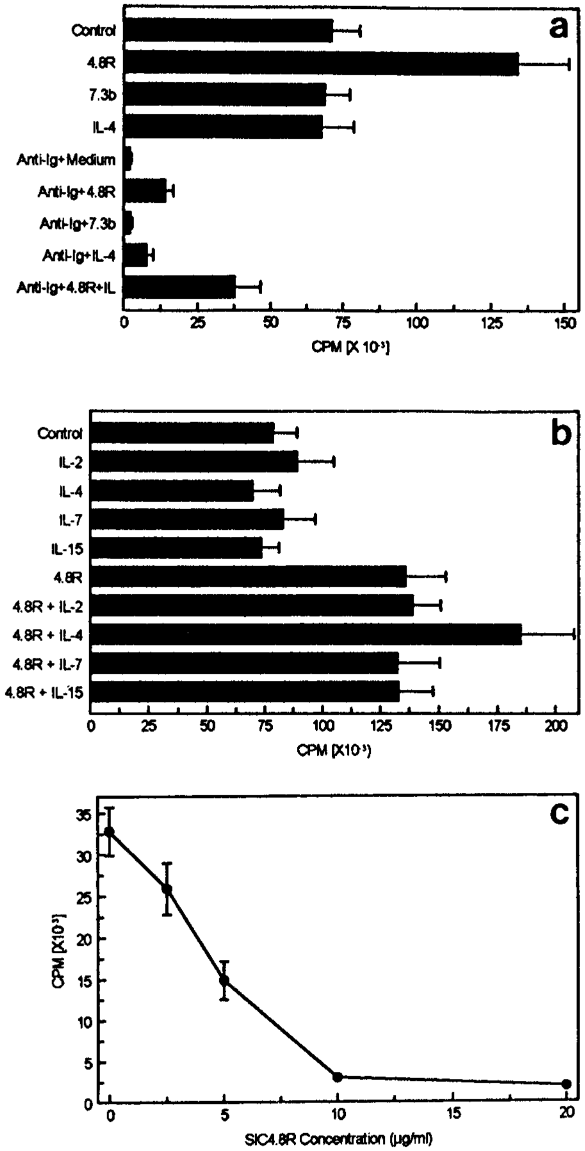

FIGURE 7. Soluble SIC4.8R altered the proliferative responses of both iPfB cells and murine pro-B cells. (a) SIC4.8R (4.8R) induced an iPfB cell proliferative response that was not observed with an isotype-matched mouse mAb (7.3b). F(ab')$_2$ rabbit anti-sheep IgM (anti-Ig + medium)-inhibited iPfB cell proliferative responses but the addition of soluble SIC4.8R (anti-Ig + 4.8R) reduced the level of anti-Ig inhibition. The addition of both IL-4 and SIC4.8R (anti-Ig + 4.8R + IL) reduced the anti-mitotic activity of anti-Ig by approximately 50%. Proliferative responses were assayed with 4 × $10^5$ iPfB cells/well and [$H^3$]-thymidine was added 4 hr prior to harvesting the 48-hr culture. (b) SIC4.8R specifically induced IL-4 responsiveness but no response was observed with other IL-2 receptor, γ-chain-common family cytokines, including IL-2, IL-7, and IL-15. No increase in iPfB cell proliferative responses was induced by the cytokines in the absence of SIC4.8R. Proliferative responses were assayed with 4 × $10^5$ iPfB cells/well and [$H^3$]-thymidine was added 4 hr prior to harvesting the 48-hr culture. (c) Soluble SIC4.8R inhibited the proliferation of the IL-7-dependent and stromal cell-dependent mouse pro-B cell line. This anti-mitotic activity was dose-dependent, requiring 10 µg/ml purified mAb to effect maximal inhibition (>90%). Proliferative responses were assayed with 5 × $10^4$ pro-B cells and 1 × $10^4$ γ-irradiated PA-6 cells/well and 10 ng/ml recombinant human IL-7. The [$H^3$]-thymidine was added 4 hr prior to terminating the 24-hr culture.

SIC4.8R complex induced a signal(s) in iPfB cells that partially inhibited the cell death induced by cross-linking sIgM. Significant, IL-4 activity in this assay was only observed when iPfB cells were co-stimulated with SIC4.8R and further assays confirmed that soluble SIC4.8R mAb specifically induced IL-4-responsiveness but not responsiveness to several other γ-chain-common cytokines (Fig. 7b).

Finally, the functional activity of the SIC4.8R complex was investigated with the RAG2 gene-deleted/*bcl*-2 transgenic mouse pro-B cell line during co-culture with PA-6 stromal cells and in the presence of IL-7. We observed a clear dose-dependent inhibition of pro-B cell growth with soluble, purified SIC4.8R mAb (Fig. 7c). At concentrations exceeding 10 μg/ml, the SIC4.8R inhibited 90% of pro-B cell proliferation. A similar inhibition was also observed when pro-B cells were pre-incubated with SIC4.8R mAb prior to co-culture but did not occur when the PA-6 stromal cells were pre-incubated with the mAb (data not shown). For these experiments, the cells were washed three times with culture medium following pre-incubation with the mAb. In contrast, a purified, isotype-matched antibody, SIC7.3b mAb, did not alter the proliferative response of pro-B cell cultures (data not shown). Thus, SIC4.8R binding to pro-B cells resulted in a strong anti-proliferative effect. The pro-B-cell line was derived from a *bcl*-2 transgenic animal and in the presence of SIC4.8R mAb, no cell death was observed and viable pro-B cell number remained constant (data not shown).

### IPfB Cell Differentiation Downregulates Expression of the SIC4.8R Complex

SIC4.8R labeling of mouse pro-B cells, human pre-B cells, sheep cortical thymocytes, and iPfB cells suggested that this molecular complex was expressed by early precursor and immature lymphocytes. Therefore, we investigated whether a signal, such as CD40 ligand, which induces iPfB cell differentiation (Griebel and Ferrari, 1995), would alter expression of the SIC4.8R complex. SIC4.8R labeling was analyzed at 48-hr intervals following iPfB cell co-culture with mCD40L/J558L cells. During this co-culture, there was a progressive decrease in the level of SIC4.8R labeling such that after 96 hr, little detectable labeling remained (Fig. 8). Thus, CD40 signaling, which is associated with T cell-dependent B cell development, downregulated the expression of the SIC4.8R molecular complex on iPfB cells. Previous work clearly demonstrated that CD40 signaling inhibited iPfB cell death and supported a limited degree of cell-proliferation and differentiation (Fig. 6B; Griebel and Ferrari, 1995). Thus, it was unlikely that the decrease in SIC4.8R labeling resulted from the outgrowth of a minor subpopulation of sIgM$^+$SIC4.8R$^-$ B cells.

### DISCUSSION

Previous investigations of surface molecules expressed by sheep PP B cells used antibodies that reacted with lymphocytes in blood and other non-PP lymphoid tissues (Hein et al., 1989; Griebel et al., 1992a, 1992b). The present investigation took a very different approach by first selecting mAbs with a reactivity unique to PP B cells. This resulted in the selection of the SIC4.8R mAb (Fig. 1; Table 1) that displayed a remarkable cross-reactivity with a similar molecular complex on a murine pro-B cell line (Figs 4 and 5) and also reacted with human pre-B cells (Table 2). The lack of SIC4.8R reactivity with sIgM$^+$ B cells isolated from several lymphoid tissues of sheep (Table 1) and blood and bone marrow of humans (Table 2) clearly indicated that the molecular complex identified was distinct from molecules known to be expressed on mature, sIgM$^+$ B cells. Co-staining of a RAG2 gene-deleted/*bcl*-2 transgenic mouse pro-B cell line with SIC4.8R mAb and antibodies for several known pro-B cell molecules provided further evidence that a novel molecular complex had been identified (Fig. 4; data not presented). Protein chemistry confirmed that the SIC4.8R mAb identified a structurally similar molecular complex on both sheep and murine B cells (Fig. 5) and a review of known CD molecules did not reveal any B cell molecules with a similar structure or pattern of expression (Barclay et al., 1993). Thus, the selection method used to identify the SIC4.8R mAb was an effective means to detect surface molecules unique to the immature B cells present in sheep PP (Figs 1, 3, and 4).

The second objective of this investigation was to identify molecules that may function to regulate the development of PP B cells. Evidence that the SIC4.8R complex may influence iPfB cell development was provided by a number of *in vitro* assays with soluble, purified mAb (Figs 6 and 7). These assays confirmed that the SIC4.8R complex could transduce a signal(s) that altered iPfB cell survival and proliferation, co-signaling by sIgM, and induce cytokine responsiveness. The multiplicity of effects observed with soluble SIC4.8R mAb may have several explanations. This protein complex may utilize a signaling pathway that is shared by many other surface molecules. Alternatively, as suggested by the protein chemistry (Fig. 5), this molecular complex may associate with other proteins that possess co-signaling functions that can influence iPfB cell development. IPfB cell responses to the SIC4.8R mAb also raised questions regarding a possible ligand(s) for the SIC4.8R complex. It was thought that the well-defined growth requirements of the mouse pro-B cell line might provide a better system to investigate this question (Grawunder et al., in press). The antimitotic activity of soluble SIC4.8R mAb suggested that this mAb may block an essential interaction between pro-B cells and a molecule expressed on PA-6 stromal cells (Fig. 7c). However, it may actually be the absence of a ligand for the SIC4.8R complex on PA-6 cells that permits the long-term culture of mouse pro-B cells. In conclusion, the *in vitro* activity of soluble SIC4.8R mAb confirmed that this molecular complex could influence iPfB cell growth by modulating a variety of B cell responses. However, the present experiments provided no clues regarding the nature of a relevant ligand(s) for the SIC4.8R complex.

Expression of the SIC4.8R complex on precursor and immature lymphocyte populations raises further questions regarding possible developmental similarities among these different cell populations. The disparate responses of iPfB cells and the murine pro-B cell line to SIC4.8R mAb may indicate that this molecular complex performs different functions during different stages of B cell development. However, these results are preliminary and the transgenic murine pro-B cell line may not reflect normal physiological responses. One possible developmental similarity among mouse and human precursor B cells and iPfB cells may be that all these B cell development stages are stromal cell-dependent and T cell-independent (reviewed in Rolink and Melchers, 1993; Griebel and Ferrari, 1994). Previous investigations revealed that T cell-dependent B cell development predominates in the jejunal PP of lambs but plays a minor role in ileal PP B cell development (Hein et al., 1989; Griebel and Ferrari, 1995). Thus, the postnatal decline in SIC4.8R expression on jejunal PP B cells and the CD40L-induced downregulation of the SIC4.8R complex (Fig. 8) support the conclusion that the SIC4.8R complex may play a role during T cell-independent B cell development. If this conclusion is valid, then SIC4.8R labeling may reveal that T cell-independent B cell development occurs in all fetal PP but this pathway of B cell development declines in the jejunal PP of lambs (Figs 1 and 3). Furthermore, the absence of SIC4.8R$^+$ lymphoid cells in bone marrow (Fig. 4) may reveal that this is not an active site for B lymphopoiesis in

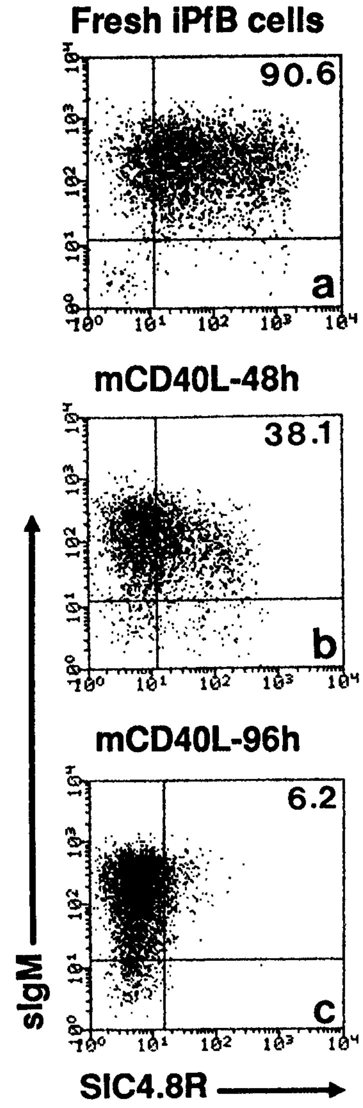

FIGURE 8. Co-culture of iPfB cells with mCD40L/J558L cells downregulated SIC4.8R expression. (a) Freshly isolated sIgM$^+$ iPfB cells labeled with SIC4.8R. (b) After a 48-hr co-culture with mCD40L/J558L cells, fewer than 40% sIgM$^+$ iPfB cells labeled with SIC4.8R. (c) Following a 96-hr co-incubation with mCD40L/J558L cells, fewer than 10% sIgM$^+$ iPfB cells labeled with SIC4.8R. All phenotypic analyses were restricted to viable cells (PI$^-$ FALS$^{hi}$), and viable iPfB cell number remained relatively constant throughout the 96-hr culture period. The percent SIC4.8R$^+$ sIgM$^+$ B cells is indicated in quadrant 2 of each profile.

young lambs. There is presently no direct evidence that the SIC4.8R complex is conserved on sheep pre-B cells but investigations are now in progress to further characterize the population of sIgM$^-$SIC4.8R$^+$ cells observed in the fetal PP (Figs 3a and b). If these cells are indeed pre-B cells, then this may provide substantial evidence that sheep possess a pathway of B cell development in the PP that is distinct from that defined for rodents (reviewed in Rolink and Melchers, 1993).

Identification of the SIC4.8R complex provided further evidence that the B cell population present in sheep PP is distinct from B cells in other lymphoid tissues and provided further insights into the developmental biology of sheep PP B cells. Conservation of the SIC4.8R protein complex on precursor and immature lymphocytes of both the T and B cell lineage in several mammalian species suggests that this molecular complex may play an important role or roles in regulating lymphopoiesis. The *in vitro* assays with soluble SIC4.8R mAb clearly demonstrated that this molecular complex can transduce signals that influenced iPfB cell and mouse pro-B cell growth and survival. Therefore, further characterization of the structure and function of this complex should provide significant insights into the regulation of mammalian lymphocyte development.

### MATERIALS AND METHODS

### Generating and Selecting Hybridomas

BALB/c mice were injected intraperitoneally, 3 times at monthly intervals, with iPfB cells isolated from 6–8-week-old lambs. Three days after the last injection of iPfB cells, the splenocytes were fused with Sp 2/0 myeloma cells using standard procedures. Hybridomas were then screened for reactivity with sheep iPfB cells, but no reactivity with peripheral blood mononuclear (PBM) cells using a protocol described by Davis et al. (1995). Briefly, PBM cells were prelabeled with hydroethidine (Polysciences, Warrington, PA) and then mixed 1:1 with iPfB cells. Thus, during flow cytometric analyses, the PBM cells could be distinguished from iPfB cells by their fluorescence in FL2. An aliquot of the PBM cell and iPfB cell mixture was then incubated with culture supernatant from each hybridoma, washed three times with phosphate-buffered saline (PBS), and reacted with FITC-goat anti-mouse Ig (Southern Biotechnology Associates, Birmingham, AL). Samples were analyzed with a FACScan flow cytometer (Becton-Dickinson, Mountain View, CA) and mAbs that reacted with iPfB cells but not PBM cells were further screened in an immunohistochemical (IHC) stain of cryosections prepared from ileal PP, jejunal PP, thymus, and mesenteric lymph node (MLN). All clones selected by flow cytometric analysis were subcloned by limiting dilution and a second subcloning was performed following selection by immunohistochemistry.

### Isolation of Lymphoid Cells

All experiments were conducted using 6–10-week-old suckling lambs or 142–144-day gestation fetuses (term = 150-day gestation) of either sex from Versuchbetrieb Sennweid (Olsburg, Switzerland). Blood was collected in EDTA and PBM cells were isolated with a discontinuous Percoll gradient, as described previously (Dudler et al., in press). Single-cell suspensions for mouse immunizations, flow cytometry, and cultures were prepared from lymphoid follicles of the ileal and jejunal PP of lambs, fetuses, and 1–2-week-old calves, as described previously (Griebel, in press). Single-cell suspensions were prepared from thymus and MLN by mincing tissues in PBS and then filtering the cell suspension through a 20-μm nylon mesh (Small Parts, Miami, FL). Bone marrow cells were isolated from the sternum of lambs, as described previously (Haig, in press). Heparinized samples of human bone marrow were obtained by iliac crest aspiration, following institutional guidelines of the Basel Kantonspital and Kinderspital of Basel. Bone marrow aspirates were from 4 children, two boys and two girls between 3 and 11 years of age, who were determined to be healthy and free of hematological diseases. Human blood was obtained from 3 clinically normal donors and the buffy coat collected. Human bone marrow cells and PBM cells were isolated by centrifugation over a Ficoll-Hypaque density gradient (1.077 g/ml; Pharmacia, Uppsala, Sweden).

### Flow Cytometry and Immunohistochemistry

All analyses were conducted with either a FACScan, FACStar Plus, or FACSVantage (Becton-Dickinson). Indirect immunofluorescence of single- or dual-labeled sheep cells was completed with unconjugated mAbs that were detected with isotype-specific, FITC-, or PE-conjugated goat anti-mouse Ig (Southern Biotechnology), as described previously (Griebel and Ferrari, 1994, 1995). The anti-sheep IgM

mAb (PIg45A) was purchased from VMRD (Pullman, WA) and the anti-sheep CD5 mAb (ST1a) was produced from a hybridoma maintained at the Basel Institute for Immunology. The anti-mouse heat-stable antigen (HSA; J11d), anti-mouse CD25 (7D4), and anti-mouse CD43 (S7) were purchased from Pharmingen (San Diego). The anti-mouse IL-7 receptor mAb (Sudo, et al., 1993) was a generous gift from S.I. Nishikawa (Kumamoto University School of Medicine, Kumamoto, Japan). Flow cytometric analysis of surface molecules on human lymphocytes was done by direct and indirect immunofluorescence using the following mAbs: FITC-anti-CD2; FITC-anti-CD3; PE- and biotinylated anti-C4; FITC-anti-CD7; biotinylated anti-CD8; biotinylated anti-CD10; biotinylated anti-CD19; FITC-anti-CD20; PE-anti-CD33; FITC- and PE-anti-CD34; FITC-anti-CD56; and FITC-anti-γδ TcR (Becton-Dickinson). The FITC-anti-αβ TcR mAb was purchased from T Cell Diagnostics (Cambridge, MA). The FITC-conjugated, $F(ab')_2$ rabbit anti-human IgM was purchased from Dakopatts (Glostrup, Denmark) and the PE-conjugated goat anti-mouse IgM and PE- and TRI-streptavidin were purchased from Southern Biotechnology. The bone marrow cells were maintained at 4°C during the labeling procedure and were analyzed within 4 hr of aspiration. All flow cytometric analyses were restricted to viable cells by adding 2 μg/ml propidium iodide (PI; Calbiochem-Behring, La Jolla, CA) to each sample and excluding all positive cells in FL3. Identification of apoptotic and viable ileal PP B cells was based on an analysis of both PI staining and forward angle light scatter (FALS; Griebel and Ferrari, 1995). The preparation of sheep tissues for cryosections and the indirect immunoperoxidase method to detect mAb binding in tissue sections were as previously described in detail (Griebel et al., 1996).

### Protein Chemistry

Freshly isolated ileal PP cells or cultured murine pro-B cells were washed three times with PBS and then surface biotinylated with 0.2 mg/ml Sulfo-NHS-biotin (Pierce Chemical, Rockford, IL). After 20–30 min, the reaction was quenched with 25 mM lysine and cells were lysed in either NP-40 (0.5%; Sigma, St Louis, MO), digitonin (1%; Sigma), or Brij 97 (1%; Sigma). The lysis buffer contained a mixture of protease inhibitors that included leupeptin, aprotinin, amino-caproic acid, and p-aminobenzamidine (Sigma). The lysate was precleared by incubating for 2 hr with 40 μl of Seph-CNBr-goat-anti-mouse Ig beads (Pharmacia). The lysate was then reacted for 2 hr with 20 μg of mAb and the immunoprecipitation performed with Seph-CNBr beads conjugated with either goat-anti-mouse Ig (Cappel) or goat anti-mouse IgM (Southern Biotechnology). After 3 washes, the beads were boiled in Laemli sample buffer (±DTT) before running on a discontinuous SDS-PAGE. Proteins were transferred by semidry blot onto Immobilon PVDF (Millipore) and the filter was then blocked with nonfat dry milk (5% in PBS with 0.1% Tween-20). The filter was washed, reacted with Strep-HRPO (1:5000; Southern Biotechnology), and then developed with ECL Detection reagents (Amersham, Buckinghamshire, UK) and Hyperfilm-ECL (Amersham).

### Cell Lines

A murine pro-B cell line, carrying all Ig genes in germline configuration, was established from the fetal liver of a RAG-2 gene-deleted/*bcl*-2 transgenic mouse (Grawunder et al., in press). The sustained growth of this cell line was dependent on contact with PA-6 stromal cells and exogenous IL-7. J558L cells were transfected with a plasmid expressing the mouse CD40L gene, and L-Histidinol (Sigma) was used to select a clone that expressed high levels of membrane CD40L (mCD40L; Peter Lane, Basel Institute for Immunology). Prior to co-culture, the mCD40L/J558L cells were γ-irradiated (9000 rads) to inhibit cell growth. This irradiation did not reduce mCD40L expression and mCD40L was previously shown to interact specifically with CD40 expressed on iPfB cells (Griebel and Ferrari, 1995).

### Cell Cultures

All cultures were incubated at 37°C in a humidified atmosphere with 7% $CO_2$ in air. The culture medium was Iscove's modified Dulbecco's modified Eagle medium (Gibco/BRL, Basel) supplemented as previously described (Griebel et al., 1993) plus 2% FBS (Gibco/BRL). Cultures for the analysis of viable cell number and cell phenotype were conducted in 6-well plates (Nunc, Roskilde, Denmark) and proliferative response assays were conducted in flat-bottom, 96-well plates (Nunc). Investigations of SIC4.8R and SIC7.3b (IgM isotype) activity with cultured cells were conducted with ammonium-sulfate-purified mAb and SIC4.8R mAb was more active in solution than bound to plastic. The $F(ab')_2$

rabbit anti-sheep IgM (Cappel Laboratories, West Chester, PA) was used at a final concentration of 40 μg/ml (Griebel et al., 1991). The recombinant human IL-2, IL-4, IL-7, and IL-15 (Peprotech EC, London) were used at a final concentration of 100 ng/ml and had specific cross-species activities with iPfB cells (Griebel and Ferrari, manuscript submitted). The mCD40L/J558L cells were cocultured with iPfB cells at a final ratio of 1:10 (mCD40L:iPfB cells). IPfB cells were cultured at a density of $10 \times 10^6$ cells/well in 6-well plates and $4 \times 10^5$ cells/well in 96-well plates. The murine pro-B cells were cultured in 96-well plates at a density of $5 \times 10^4$ pro-B cells with $1 \times 10^4$ γ-irradiated PA-6 cells/well and 10 ng/ml recombinant human IL-7. Proliferative response assays were conducted in quadruplicate and incubated for 48 hr with 1 μCi [$H^3$]-thymidine (Amersham) added during the last 4 hr of culture. The incorporation of [$H^3$]-thymidine was determined using standard methods with a Betaplate 96-well Harvester and Betamax liquid scintillation counter (LKB Wallac, Turku, Finland).

### ACKNOWLEDGMENTS

We gratefully acknowledge the assistance of Dr Alois Gratwohl, Division of Hematology, Dept. of Research, Kantospital Basel, Switzerland, and Dr Erich Signer, Dept. of Pediatric Hematology and Oncology, Kinderspital, Basel, Switzerland, in providing samples of human bone marrow. We thank Drs W.R. Hein and B.A. Imhof for their critical review of the manuscript. The Basel Institute for Immunology was founded and is supported by F. Hoffman LaRoche & Co. Ltd., Basel, Switzerland.

*(Received January 25 1996)*

*(Accepted 1996)*

### REFERENCES

Barclay A.N., Birkeland M.L., Brown M.H., Beyers A.D., Davis S.J., Somoza C., and Williams A.F. (1993). The leucocyte antigen factsbrook (London, Academic Press), pp. 102–412.

Chapman H.A., Johnson J.S., and Cooper M.D. (1974). Ontogeny of Peyer's patches and immunoglobulin-containing cells in pigs. J. Immunol. **112**: 555–563.

Cooper M.D., and Lawton A.R. (1972). The mammalian "Bursa equivalent": Does lymphoid differentiation along plasma cell lines begin in the gut-associated lymphoepithelial tissues (GALT) of mammals? Contemp. Topics Immunobiol. **1**: 49–68.

Davis W.C., Davis J.E., and Hamilton M.J. (1995). Use of monoclonal antibodies and flow cytometry to cluster and analyze leukocyte differentiation molecules. *In*: Monoclonal antibody protocols, Davis W.C., Ed. (Clifton, NJ: Humana Press), pp. 149–167.

Dudler L., Griebel P.J., and Hein W.R. Separation of mononuclear cells from sheep blood. *In*: Immunological methods manual, Lefkovitts I., Ed. (London: Academic Press).

Grawunder U., Rolink A., and Melchers F. (1955) Induction of sterile transcription from the kL chain gene locus in V(D) J recombinase deficient progenitor B cells. Int. Immunol. 7: 1915–1925.

Griebel P.J. (In press). Isolation of lymphoid follicles from Peyer's patches. *In*: Immunological methods manual, Lefkovitts I., Ed. (London: Academic Press).

Griebel P.J., Davis W.C., and Reynolds J.D. (1991). Negative signaling by surface IgM on B cells isolated from ileal Peyer's patch follicles of sheep. Eur. J. Immunol. **21**: 2281–2284.

Griebel P.J., Davis W.C., and Reynolds J.D. (1992b). An analysis of the growth and differentiation of B cells isolated from follicles of the ileal Peyer's patch of sheep. Immunol. **75**: 601–607.

Griebel P.J., and Ferrari G. (1994). Evidence for a stromal cell-dependent, self-renewing B cell population in lymphoid follicles of the ileal Peyer's patch of sheep. Eur. J. Immunol. **24**: 401–409.

Griebel P.J., and Ferrari, G. (1995). CD40 signaling in ileal Peyer's patch B cells: Implications for T cell-dependent antigen selection. Int. Immunol. **7**: 369–379.

Griebel P.J., Hein W.R., Dudler L., and Ferrari G. (1993). Phenotype and function of stromal cells cloned from the ileal Peyer's patch of sheep. Stem Cells **11**: 130–143.

Griebel P.J., Kennedy L., Graham T., Davis W.C., and Reynolds J.D. (1992a). Characterization of B-cell phenotypic changes during ileal and jejunal Peyer's patch development in sheep. Immunology **77**: 564–570.

Griebel P.J., Kugelberg B., and Ferrari G. (1996). Two distinct pathways of B-cell development in Peyer's patches. Develop. Immunol. **4**: 263–277.

Haig D. (In press). Haemopoietic cell culture in sheep. *In*: Immunological methods manual, Lefkovitts I., Ed. (London: Academic Press).

Hein W.R., Dubler L., and Mackay C.R. (1989). Surface expression of differentiation antigens on lymphocytes in the ileal and jejunal Peyer's patches of lambs. Immunology **68**: 365–370.

Motyka B., and Reynolds J.D. (1991). Apoptosis is associated with the extensive B cell death in the sheep ileal Peyer's patch and the chicken bursa of Fabricius: A possible role in V cell selection. Eur. J. Immunol. **21**: 1951–1958.

Reynaud C.-A., Garcia C., Hein W.R., and Weill J.-C. (1995). Hypermutation generating the sheep immunoglobulin repertoire is an antigen-independent process. Cell **80**: 115–125.

Reynolds J.D. (1986). Evidence of extensive lymphocyte death in sheep Peyer's patches. I. A comparison of lymphocyte production and export. J. Immunol. **136**: 2005–2010.

Reynolds J.D. (1987). Mitotic rate maturation in the Peyer's patches of fetal sheep and in the bursa of Fabricius of the chick embryo. Eur. J. Immunol. **17**: 503–507.

Reynolds J.D., and Morris B. (1983). The evolution and involution of Peyer's patches in fetal and postnatal sheep. Eur. J. Immunol. **13**: 627–635.

Rolink A., and Melchers F. (1993). Generation and regeneration of cells of the B-lymphocyte lineage. Curr. Opinion Immunol. 5: 207–217.

Sudo T., Nishikawa S., Ohno N., Akiyama N., Tamakoshi M., Yoshida H., and Nishikawa S.-I. (1993). Expression and function of the interleukin 7 receptor in murine lymphocytes. Proc. Natl Acad. Sci. USA **90**: 9125–9129.

